# Identification and Characterization of *Eimeria tenella* Rhoptry Protein 35 (EtROP35)

**DOI:** 10.3390/vetsci9090465

**Published:** 2022-08-29

**Authors:** Bingxiang Wang, Ningning Zhao, Jinkun Sun, Lingyu Sun, Huihui Li, Zhiyuan Wu, Hongmei Li, Xiao Zhang, Xiaomin Zhao

**Affiliations:** 1 Department of Preventive Veterinary Medicine, College of Veterinary Medicine, Shandong Agricultural University, Tai’an 271002, China; 2 Shandong Provincial Key Laboratory of Animal Biotechnology and Disease Control and Prevention, Shandong Agricultural University, Tai’an 271002, China; 3 Shandong Provincial Engineering Technology Research Center of Animal Disease Control and Prevention, Shandong Agricultural University, Tai’an 271002, China

**Keywords:** *Eimeria tenella*, EtROP35, immunological protection, rhoptry protein

## Abstract

**Simple Summary:**

Rhoptry proteins (ROPs) of phylum Apicomplexa parasites are important secretory virulence factors as well as candidate vaccines. However, studies on ROPs of *Eimeria tenella* are limited. In this study, the coding sequence of *E. tenella* rhoptry protein 35 (EtROP35) was cloned, and then its localization, expression in parasite, potential role within invasion, and protective efficacy were investigated. Sequence analysis and subcellular localization revealed that EtROP35 is a rhoptry protein of *E. tenella*. Sporozoite invasion-blocking assay and protective efficacy indicated that EtROP35 might be involved in the parasite invasion process and may be a potential vaccine candidate against *E. tenella*.

**Abstract:**

Rhoptry proteins (ROPs) of Apicomplexa are crucial secreted virulence factors and sources of vaccine candidates. To date, *Eimeria tenella* ROPs are not well studied. This study identified and characterized a novel *E. tenella* ROP (EtROP35), which showed the highest levels among 28 putative ROPs in previous sporozoite and merozoite transcriptomes. Sequence analysis showed that EtROP35 contains an N-terminal secretory signal and a protein kinase domain including eight conserved ROP35-subfamily motifs. Subsequent experiments confirmed that it is a secretory protein. Subcellular localization revealed it localized at the apical end of the sporozoites and merozoites, which was consistent with the ROPs of other Apicomplexan parasites. To further understand the biological meaning of EtROP35, expression levels in different developmental stages and sporozoite invasion-blocking assay were investigated. EtROP35 showed significantly higher levels in sporozoites (6.23-fold) and merozoites (7.00-fold) than sporulated oocysts. Sporozoite invasion-blocking assay revealed that anti-EtROP35 polyclonal antibody significantly reduced the sporozoite invasion rate, suggesting it might participate in host cell invasion and be a viable choice as a vaccine candidate. The immunological protective assays showed that EtROP35 could induce a high level of serum IgY and higher mean body weight gain, and lower cecum lesion score and oocysts excretion than the challenged control group. These data indicated that EtROP35 had good immunogenicity and may be a promising vaccine candidate against *E. tenella*.

## 1. Introduction

*E. tenella* is a highly destructive and virulent pathogen threatening global poultry industry [1]. It belongs to the phylum Apicomplexa, which includes notable species such as *Toxoplasma*, *Plasmodium*, *Eimeria, Babesia*, *Neospora* and *Cryptosporidium*. As an obligate intracellular apicomplexan protozoan parasite, the infection and intracellular development of *E. tenella* rely on three unique apical secretory organelles: rhoptries, micronemes, and dense granules. Rhoptries discharging ROPs are club-shaped organelles divided into two distinct substructures (posterior bulb and anterior neck) [2,3]. ROPs are the most crucial secreted virulence factors and play critical roles in host cell invasion, parasitophorous vacuole formation, and regulation of host cells [4,5,6].

Due to the presence of protein kinase domains, ROPs are specific members of eukaryotic protein kinases (ePK) [7]. Among the 90 ePK genes found in the *E. tenella* genome, 28 are likely to be ROPs [8]. To date, four *E. tenella* ROPs have been reported, including EtROP1, Et-ROPK-Eten5-A, EtROP17 and EtROP30 [9,10,11,12]. EtROP1 was expressed and identified in *Toxoplasma gondii* [9]. Et-ROPK-Eten5-A and EtROP17 performed well as vaccine candidates [10,11]. EtROP30 could enter host nucleus and might be an important virulence factor [12]. ROPs of *T. gondii* have been widely studied since tachyzoite can be continuously propagated in vitro and a set of powerful genetic manipulation and functional analysis tools can be applied, some ROPs (such as ROP5, ROP16 and ROP18) have been proved to be key virulence factors and control targets. In contrast, studies on *E. tenella* ROPs were limited, possibly due to in-vitro culturing and genetic manipulation difficulties.

A previous RNA-Seq analysis of sporozoite and merozoite transcriptomes found the highest concentration of EtROP35 among 28 putative ROPs [13]. This observation suggested its possible critical role during the infectious stages. Furthermore, EtROP35 is homologous to the *T. gondii* rhoptry protein 35 (TgROP35) in the evolutionary relationship analysis. TgROP35 strongly stimulates the host immune response and is considered a promising drug target and vaccine candidate antigen [14]. Here, EtROP35 was identified, and its protein properties, localization, and expression levels during different developmental stages were investigated. Moreover, its roles in sporozoite invasion and immunological protection against *E. tenella* were evaluated.

## 2. Materials and Methods

### 2.1. Parasites and Animals

*E. tenella* separated from Shandong (SD-01 strain) was propagated in our laboratory [15]. Unsporulated oocysts, sporulated oocysts, sporozoites, and second-generation merozoites were obtained according to the published methods [16]. The Dong-Yue Breeding Poultry Company Limited (Tai’an, China) supplied one-day-old Hyline Brown cocks and raised them in an environment free of coccidiosis. The study protocol and animal experiments associated were approved by Animal Care and Use Committee of Shandong Agricultural University (ID: SDAUA-2021-046).

### 2.2. Molecular Cloning and Bioinformatic Analysis of EtROP35

The coding sequence of EtROP35 gene was cloned from *E. tenella* sporozoite cDNA by PCR (primers: EtROP35-F: 5′-ATGAGCCTGCGCGCC-3′; EtROP35-R: 5′-CAGGAACTTGTAGGGAGTCTGG-3′). Bioinformatic analyses were performed using ExPASy (http://www.expasy.org/, accessed on 10 March 2020). All EtROP35 homology protein sequences from *Eimeria* species were downloaded from the NCBI database (https://www.ncbi.nlm.nih.gov/, accessed on 16 March 2020): *E. tenella* (XP_013228816.1), *E. necatrix* (XP_013434354.1), *E. brunetti* (CDJ51900.1), *E. acervulina* (XP_013247055.1), *E. maxima* (XP_013335700.1), *E. praecox* (CDI74752.1), and *E. mitis* (XP_013358067.1).

### 2.3. Localization of EtROP35

The recombinant EtROP35 protein was expressed in *E. coli* BL21 carrying a pET32a-EtROP35 plasmid and purified by His-tag Protein Purification (Solarbio Science & Technology Co., Ltd., Beijing, China). The anti-EtROP35 polyclonal antibody was generated to localize the parasites using indirect immunofluorescence assay (IFA) following a previous method [17]. Briefly, sporozoites and second-generation merozoites were fixed for 25 min with 4% paraformaldehyde. Samples were then permeabilized for 20 min with 0.25% Triton X-100. The coverslips were blocked in 3% BSA at 37 °C for 1 h. Anti-EtROP35 mouse polyclonal antibody (1:150) was used to incubate the parasites overnight at 4 °C. A FITC-labeled second antibody (Proteintech Wuhan Sanying, Wuhan, China) was used to label according to the manufacturer’s instructions. Fluorescence microscopy (Olympus, Tokyo, Japan) was adopted for capturing images under the oil objective.

### 2.4. Sporozoite Secretion Experiment

According to a previous method [18], sporozoites were incubated at 37 °C for 15 min in the presence of 400 nM calcium ionophore A23187 (Sigma, St. Louis, MO, USA). The supernatant (secretory protein) and sporozoite lysate were analyzed using Western blot by using the anti-EtROP35 polyclonal antibody (1:1000). Anti-GAPDH mouse monoclonal antibody (Solarbio Science & Technology Co., Ltd., Beijing, China) as the reference control (1:6000), and HRP-conjugated anti-mouse IgG (Proteintech Wuhan Sanying, Wuhan, China) as the secondary antibodies at 1:8000.

### 2.5. The Transcription and Expression Levels of EtROP35 in Different Developmental Stages of E. tenella

The transcription levels of EtROP35 in four stages (unsporulated oocysts, sporulated oocysts, sporozoites, and second-generation merozoites) of *E. tenella* were detected by employing qRT-PCR using SYBR Green I (Vazyme Biotech Co., Ltd., Nanjing, China) (primers: F: 5′-ATGAGCCTGCGCGCC-3′; R: 5′-CAGGAACTTGTAGGGAGTCTGG-3′) with the 18S rRNA as the standard reference gene (Primers: Et18S-F: 5′-TGTAGTGGAGTCTTGGTGATTC-3′; Et18S-R: 5′−CCTGCTGCCTTCCTTAGATG-3′). The translation levels of EtROP35 were detected by western blot using the anti-EtROP35 polyclonal antibody (1:1000). Parasites of four stages were ground in liquid nitrogen and the protein lysates were prepared in RIPA buffer including protease inhibitor (Solarbio Science & Technology Co., Ltd., Beijing, China). The ImageJ 1.8.0 software (National Institutes of Health, Bethesda, MD, USA) was used to measure Grey values.

### 2.6. E. tenella Sporozoite Invasion-Blocking Assay

The sporozoite invasion-blocking assay was conducted based on the observation that sporozoites invaded DF-1 (chicken embryo fibroblast cell line) cells. The sporozoites were pretreated with anti-EtROP35 serum and mouse serum at 37 °C for 2 h with multiple dilutions, separately. DF-1 cells were infected with 3 × 10^5^ sporozoites per well of a 24-well plate. After culturing for 12 h, the uninvaded free sporozoites were gathered and counted. The inhibition rates were calculated from the percentage of the uninvaded sporozoites.

### 2.7. The Immune Protection of EtROP35 Proteins

Three groups (20 chickens/group) were used to evaluate the immune protection of EtROP35.Chickens were immunized in the neck region subcutaneously with 200 µL/chicken (100 µg) purified His-EtROP35 protein in complete Freund’s adjuvant at 7-day age in EtROP35 group. The challenged and unchallenged control groups received injections of complete Freund’s adjuvant emulsified in PBS. A second immunization was given at the same dose in incomplete Freund’s adjuvant at the following week. One week later, fresh *E. tenella* sporulated oocysts were orally ingested in the EtROP35 group and the challenged control group (8000/chicken), while the unchallenged group were ingested PBS. The cecal lesion scores of the chickens (n = 5) from each group were recorded using a continuous number from 0 (none) to 4 (severe) at 5 days post-infection (dpi); the grading criteria were in line with the previous description [19]. Bodyweight gain, oocyst output, and oocyst reduction rates were calculated by the reported methods [10]. Briefly, 15 chickens in each group were measured for body weight gain between 0 dpi and 10 dpi. The number of oocysts per gram (OPG) was calculated by collecting the feces of each group between 7 dpi and 9 dpi.

### 2.8. Detection of Serum Antibody Level

The serum antibody levels (five chickens/group) were detected by ELISA at 0 and 7 dpi following reported methods [20]. Briefly, the IgY levels were detected using ELISA plates coated with purified recombinant EtROP35 protein (0.5 µg/well) and HRP rabbit anti-chicken IgY (Solarbio Science & Technology Co., Ltd., Beijing, China) as the secondary antibody. All the samples were tested in triplicates. Meanwhile, the optical density at 450 nm (OD_450_) was recorded by an automated microplate reader (Biotek Instruments Inc., Winooski, VT, USA).

### 2.9. Statistical Analysis

Statistical analysis was performed using GraphPad Prism8 (GraphPad Software, Inc., San Diego, CA, USA). Data were analyzed by the two-tailed *t*-test. The statistical significance level was expressed as *p* < 0.05.

## 3. Results

### 3.1. Identification of EtROP35

**Figure 1:**
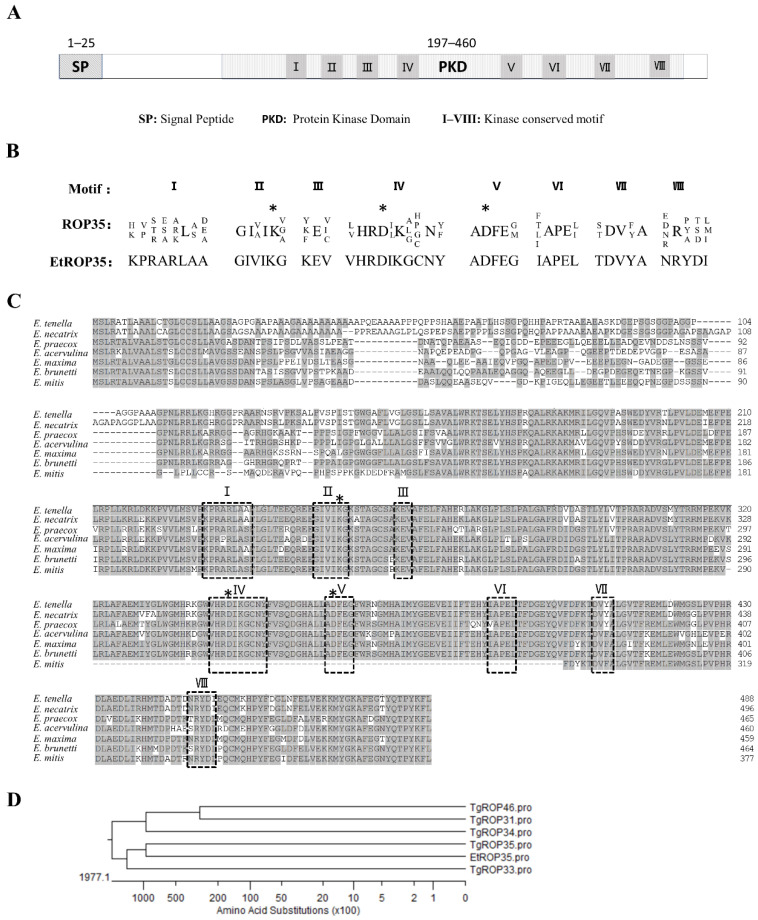
Identification and characterization of EtROP35. (**A**) Primary structure of EtROP35. (**B**) Eight conserved ROP35-subfamily motifs. (**C**) Alignment analysis of the EtROP35 homologous protein sequences in Eimeria. Conserved motifs of protein kinase (I–VIII) are framed out with dotted lines. Asterisks (*) indicate the active sites. (**D**) Evolutionary relationship analysis of EtROP35 with the homologous proteins of *T. gondii* by the MegAlign software v7.1.0 (DNASTAR Inc., Madison, WI, USA).

The cDNA sequence of EtROP35 from SD-01 strain was 1467 bp in length, and it shared 100% identity with the sequence in the GeneBank (XM_013373362.1). Bioinformatics analysis confirmed the presence of a 25-aa N-terminal secretory signal sequence and an ePK domain (197–60 aa) (Figure 1A). From a previous study, 28 deductive ROPs of *E. tenella* were divided into ten subfamilies by evolutionary adaptation and into active and inactive enzymatic ROPs based on the presence or not of the “catalytic triad” [21]. According to this criterion, EtROP35 contained eight conserved ROP35-subfamily motifs (KPRARLAA, GIVIKG, KEV, VHRDIKGCNY, ADFEG, IAPEL, TDVYA, and NRYDI) and three key residues (K^253^, D^344^, and D^362^) essential for catalysis (Figure 1B), indicating that EtROP35 was an active enzymatic ROP of the ROP35 subfamily. The EtROP35 homologous proteins from all seven species of *Eimeria* were obtained from the NCBI database using protein BLAST. Sequence alignment revealed highly conserved homologous rhoptry proteins between species, especially the protein kinase catalytic site and eight motifs. However, the last two catalytic sites and motifs IV, V, and VI were absent in *Eimeria mitis* (Figure 1C). EtROP35 showed 97.33% identity to *E. necatrix*, 87.23% identity to *E. brunetti*, 80.27% identity to *E. acervulina*, and 79.60% identity to *E. mitis*. Evolutionary relationship analysis revealed that EtROP35 was the closest to TgROP35 among the homologous protein from *T. gondii*, which is closely related to *E. tenella* in the phylum Apicomplexa (Figure 1D).

### 3.2. The Localization of EtROP35

**Figure 2:**
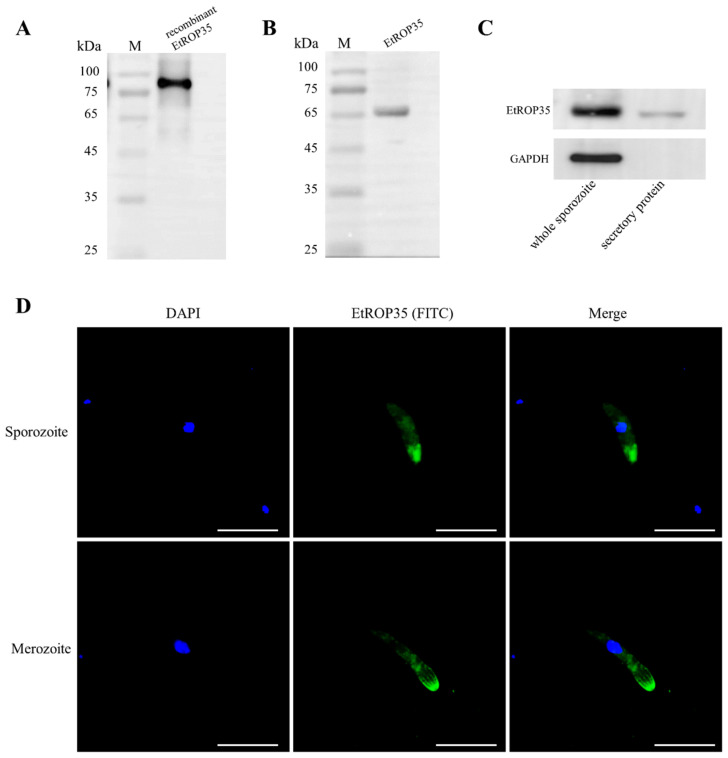
The Subcellular localization and secretion of EtROP35. (**A**) Evaluation of the recombinant EtROP35 by Western blot using the anti-EtROP35 polyclonal antibody (1:1000). (**B**) Investigation of the total sporozoite protein by Western blot through the anti-EtROP35 polyclonal antibody (1:1000). (**C**) Western blot analysis of the complete sporozoite protein and the supernatant (secretory protein). GAPDH is used as the reference. (**D**) Localization of EtROP35 in sporozoite and merozoite is visualized by IFA. Scale bar, 20 µm. (Full Western Bolt is in Supplementary Materials, Figure S1A–C).

Recombinant His-tag EtROP35 protein was expressed and purified by His bind purification Kit (TransGen Biotech), we prepared anti-EtROP35 polyclonal antibody to recognize EtROP35. The polyclonal antibody could recognize the recombinant EtROP35 protein (Figure 2A). A unique band was evident in the western blot analysis of the total sporozoite protein (Figure 2B), indicating that the anti-EtROP35 polyclonal antibody specifically recognized the native protein of the parasites. The sporozoite secretion experiment identified EtROP35 in the supernatant in which the sporozoites were incubated. At the same time, GAPDH (cytoplasmic reference) could not be detected (Figure 2C). This finding demonstrated that EtROP35 was derived from the sporozoite secretion rather than body lysis, and EtROP35 was a secretory protein. Immunolocalization experiments identified EtROP35 at the apical tip of the *E. tenella* sporozoites and merozoites (Figure 2D); this observation was consistent with the previous description of rhoptry proteins [22,23]. The localization and sequence features indicated that EtROP35 was a novel rhoptry protein of *E. tenella*.

### 3.3. Expression of EtROP35 during Different Developmental Stages of E. tenella

**Figure 3:**
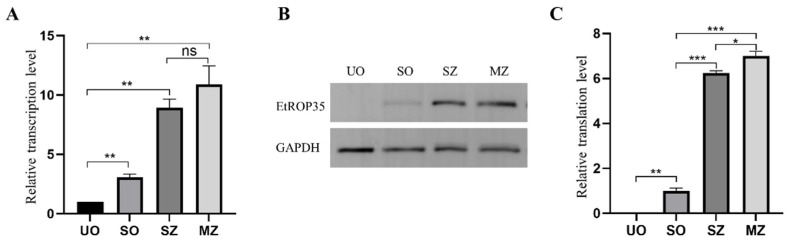
Expression levels of EtROP35 during multiple developmental stages of *E. tenella*. (**A**) Transcription levels of EtROP35. (**B**) Analysis of EtROP35 by Western blot using GAPDH as the reference. (**C**) Translation levels of EtROP35 are quantified from the Western blot bands by ImageJ 1.8.0 software (National Institutes of Health, Bethesda, MD, USA). Unsporulated oocysts (UO), sporulated oocysts (SO), sporozoites (SZ), merozoites (MZ). No obvious difference (ns): *p* > 0.05; significant difference: * *p* < 0.05, ** *p* < 0.01, *** *p* < 0.001. (Full Western Bolt is in Supplementary Materials, Figure S2).

Results of the qRT-PCR showed that transcription level of EtROP35 was higher in the sporulated oocysts, sporozoites, and merozoites (*p* < 0.01) compared to that in the unsporulated oocysts by 3.07-, 8.91-, and 10.90-fold, respectively (Figure 3A). Merozoites showed the highest transcription levels with no notable distinction from sporozoites (*p* > 0.05).

The translation levels of EtROP35 during the different developmental phases of *E. tenella* were detected through Western blot (Figure 3B). EtROP35 was not detected in the unsporulated oocysts. Its expression in the sporozoites and merozoites was higher (*p* < 0.001) compared to that in the sporulated oocysts by 6.23- and 7.00-fold (Figure 3C). Merozoites expressed the highest levels, significantly higher than sporozoites (*p* < 0.05).

### 3.4. Effect of EtROP35 on E. tenella Sporozoite Invasion of Host Cells

**Figure 4:**
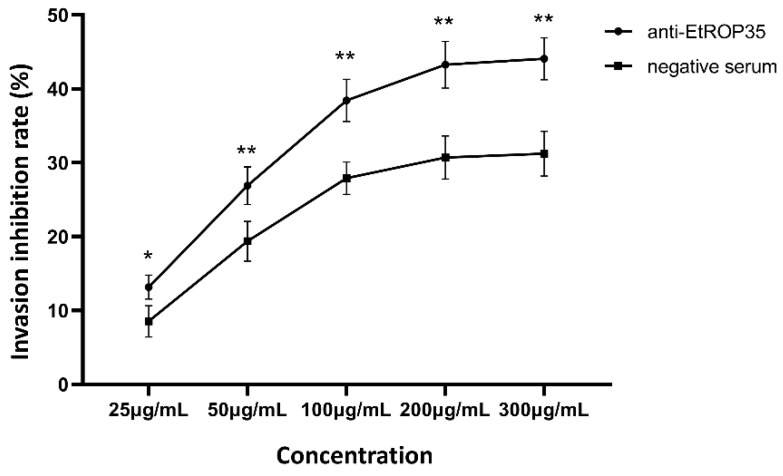
Anti-EtROP35 polyclonal antibody inhibits sporozoites invading into the host cells. Invasion inhibition rate = (uninvaded sporozoite number in the serum group/uninvaded sporozoite number in PBS group − 1) × 100%. Significant difference: * *p* < 0.05, ** *p* < 0.01.

The sporozoite invasion-blocking assay was performed to measure the impact of EtROP35 on the host cell invasion capacity of the sporozoites. Pretreatment with anti-EtROP35 polyclonal antibody significantly reduced the sporozoite invasion of the host cells (Figure 4). The invasion inhibition rates improved by increasing the antibody concentrations from 25 µg/mL to 300 µg/mL. Anti-EtROP35 polyclonal antibody blocked 44.07 ± 2.32% of the sporozoites from invading DF-1 cells at a 300 µg/mL concentration, while mouse serum blocked 31.23 ± 2.47% of the sporozoites at the same concentration (Figure 4). These findings indicated that EtROP35 might play a vital role in the progress of *E. tenella* sporozoite invasion of host cells.

### 3.5. Immune Protection Induced against E. tenella by Recombinant EtROP35

**Figure 5:**
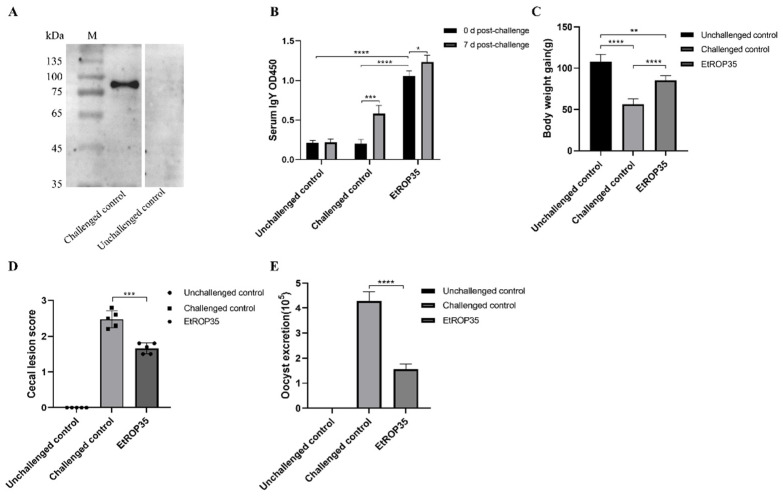
Immune protection induced by EtROP35. (**A**)The recombinant EtROP35 recognized by chicken serum. (**B**) Levels of serum IgY (OD450). (**C**) Mean body weight gain. (**D**) Cecum lesion score. (**E**) The number of oocysts excreted per gram (7 dpi to 9 dpi). Data are present as mean ±SD of three independent experiments and determined by *t*-tests. Significant difference: * *p* < 0.05, ** *p* < 0.01, *** *p* < 0.001, **** *p* < 0.0001. (Full Western Bolt is in Supplementary Materials, Figure S3).

Serum separated from challenged control group chicken was employed to assess the recombinant EtROP35 protein by western blot; the unchallenged control group served as the reference. The serum of chickens infected with *E. tenella* recognized the recombinant EtROP35 protein (Figure 5A). Thus, EtROP35 stimulated an immune response in chickens under natural infection.

Levels of IgY in serum against the EtROP35 at 0 and 7 dpi were detected by ELISA (Figure 5B). Levels of anti-EtROP35 serum antibodies in the EtROP35-immunized group at 0 dpi were significantly higher than in the unchallenged and challenged control groups (*p* < 0.0001). IgY levels were substantially higher at 7 dpi than at 0 dpi in the EtROP35-immunized group (*p* < 0.05) and the challenged group (*p* < 0.001). These results indicated that EtROP35 induced a high-level serum antibody response for *E. tenella* infection.

The indicators of protective immunity include body weight gain, cecum lesion score, OPG. The EtROP35-immunized group displayed a significant improvement (*p* < 0.0001) in the body weight gain (85.4 ± 5.3 g) compared to the challenged control group (56.2 ± 6.1 g) (Figure 5C), with the corresponding relative weight gain ratios of 78.9% and 52.0% (*p* < 0.0001). The mean cecum lesion score (1.58 ± 0.17) was significantly lower in the EtROP35-immunized group of chickens (*p* < 0.001) compared to that in the challenged control group (2.48 ± 0.21) (Figure 5D). The number of oocysts excreted was significantly reduced (*p* < 0.0001) between the EtROP35-immunized group and the challenged control group (Figure 5E), while the oocyst excretion reduction ratio was 63.4%. The results substantiated the significant immune protective effect of recombinant EtROP35 on chickens infected with *E. tenella*.

## 4. Discussion

In the current study, a novel ROP of *E. tenella* (EtROP35) was identified and characterized, showing features consistent with other ROPs of Apicomplexan members. EtROP35 was expressed at relatively higher levels during the parasite invasion stages (sporozoites and merozoites) and might participate in host cell invasion. Furthermore, EtROP35 exhibited good immunogenicity and emerged as a promising vaccine candidate.

Protein transport and secretion mechanisms are evolutionarily conserved in Apicomplexan protozoans [6]. Moreover, several typical features of ROPs, such as localization in rhoptry, secretion characteristics, and the presence of the ePK domain, are usually retained. Sequence analysis of EtROP35 demonstrated the presence of an ePK domain with eight ROP35-subfamily motifs and a “catalytic triad”. Therefore, EtROP35 is an active ROP kinase of the ROP35-subfamily. Additionally, ROPs are usually secreted from the parasite, while ePKs are typically located in the cytoplasm [7]. The EtROP35 contained an N-terminal secretory signal sequence and is secreted outside; this observation is consistent with the common characteristic of ROPs. Moreover, immunofluorescence labeled the majority of the EtROP35 on the apical end of the sporozoites and merozoites; this also is a common feature of other typical ROPs. Thus, sequence features, secretory characteristics, and localization indicated that EtROP35 is a novel ROP of *E. tenella*.

Host cell invasion is crucial for Apicomplexan parasite infection and intracellular parasitism. Studies have reported the association of ROPs with host cell invasion [24]. Nevertheless, in *E. tenella*, the reported proteins associated with host cell invasion are mainly microneme proteins [25,26], and no ROP has been experimentally proved to be involved. In the present study, the anti-EtROP35 polyclonal antibody significantly reduced the sporozoite invasion rates, indicating that EtROP35 might play a vital role in the invasion progress. In addition, differential expression of secreted proteins during the life cycle stages of parasites is generally related to their functions; higher expression levels of EtROP35 during the invasion stages support the EtROP35 association with host cell invasion.

As critical secretory virulence factors, ROPs are considered to be sources of potential vaccine candidates. Many ROPs from *T. gondii* and *Plasmodium falciparum* have emerged as hopeful vaccine candidates and achieved exciting results [27,28]. The MVA ROP2 vaccine generates an immune response, which could be helpful in protecting against toxoplasmosis [29]. Mice immunized with recombinant TgROP5 vaccine presented a prolonged survival time against the *T. gondii* RH strain [30]. TgROP16 triggers protective immune efficacy and dramatically increases the life span of mice under the challenge with the virulent RH strain of *T. gondii* [31]. In *E. tenella*, Et-ROPK-Eten5-A showed significant effects as a vaccine and was superior to Et-SAG, Et-SAG13, and Et-GRA12 [10]. EtROP17 and EtROP30 were also reported to be potential candidates for developing coccidiosis vaccines [11,12]. In addition, sporozoites and merozoites are extracellular and invasive stages of *Eimeria*. They are immunologically vulnerable and functionally vital for parasite infection. The membrane or secreted protein that enhanced expression in both stages is more conducive for an ideal vaccine [27]. In the present study, we found that EtROP35 showed higher expression during the invasion stages. Subsequent experiments showed that EtROP35 could induce a high level of serum IgY and higher mean body weight gain, lower cecum lesion score and oocysts excretion than the challenged control group, which indicated that EtROP35 had good immunogenicity and might be a promising vaccine candidate against *E. tenella*.

## 5. Conclusions

This study identified EtROP35 as a novel ROP of *E. tenella* with consistent common ROP characteristics of sequence features, secretion, and localization. In addition, EtROP35 showed higher relative expression during the invasion stages and was associated with host cell invasion. It displayed good immunogenicity and could be a promising vaccine candidate against this parasite.

## Data Availability

All data generated or analyzed during this study are included in this published article.

## Supplementary Materials

The following supporting information can be downloaded at: https://www.mdpi.com/article/10.3390/vetsci9090465/s1, Figure S1: (**A**) Evaluation of the recombinant EtROP35 by western blot using the anti EtROP35 polyclonal antibody (1:1000); (**B**) Investigation of the total sporozoite protein by western blot through the anti EtROP35 polyclonal antibody; (**C**) Western blot analysis of the complete sporozoite protein and the supernatant (secretory protein); Figure S2: Expression levels of EtROP35 during multiple developmental stages of *E. tenella* analyzed by western blot; Figure S3: The recombinant EtROP35 recognized by chicken serum from the challenged control group and unchallenged control group.
