# Supplementary material for: Identification and Characterization of Eimeria tenella Rhoptry Protein 35 (EtROP35)
Source: Vet Sci. 2022 Aug 29;9(9):465. doi: 10.3390/vetsci9090465 (PMC9505231; doi:10.3390/vetsci9090465)
Supplement: Supplementary file 1 [file vetsci-09-00465-s001.zip › vetsci-1835597-SI.pdf]

Supplementary Materials

Figure S1A

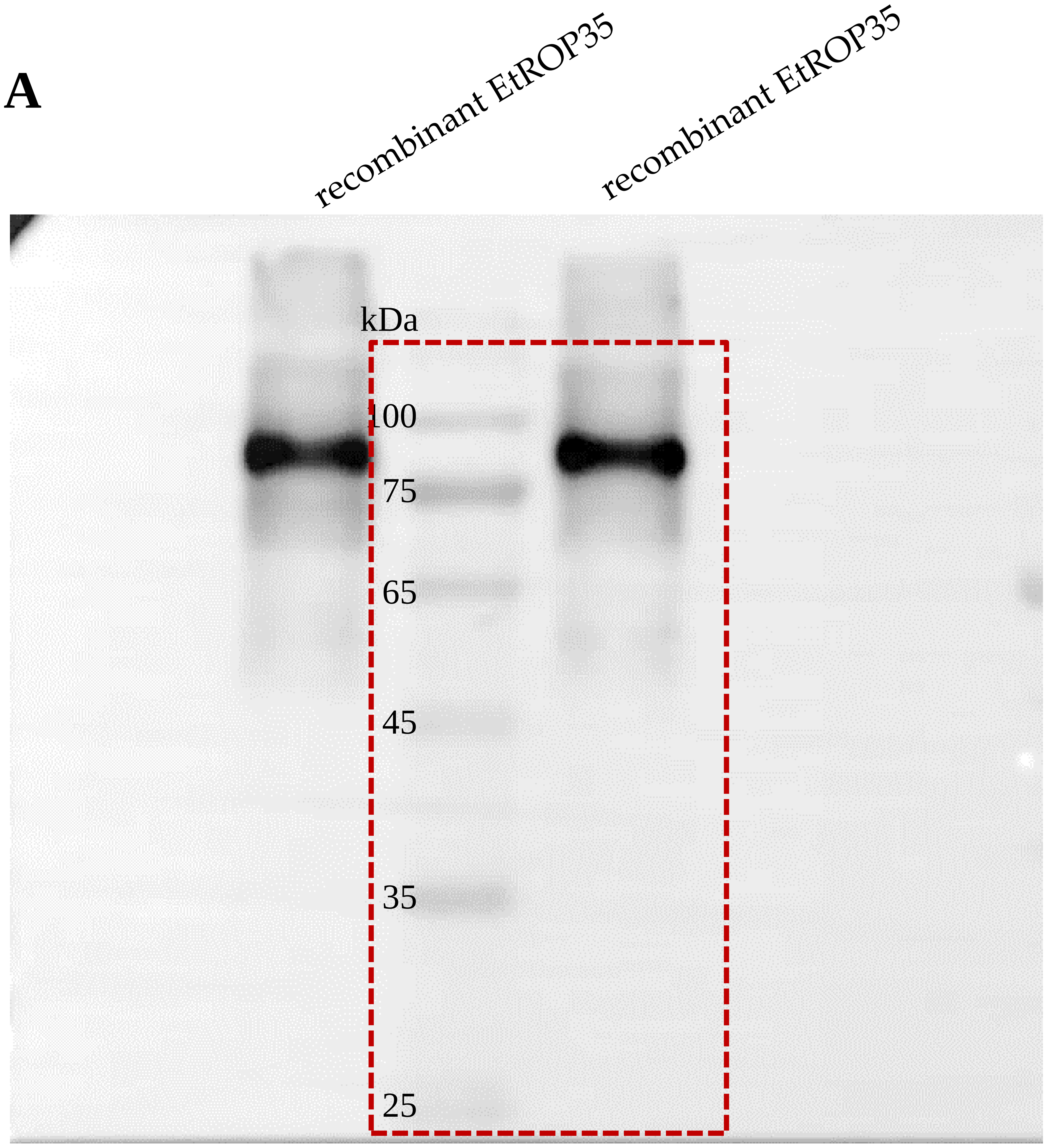

Figure S1A. Evaluation of the recombinant EtROP35 by western blot using the anti-EtROP35 polyclonal antibody (1:1000). Gel-separated proteins were transferred to polyvinylidene fluoride membranes (0.22 μm pore size; Millipore, Billerica, MA, USA). Western blots were performed using the anti-EtROP35 polyclonal antibody (1:1000). HRP-conjugated anti-mouse IgG (Proteintech Wuhan Sanying, Wuhan, China) served as the secondary antibodies at 1:8000.

Figure S1B

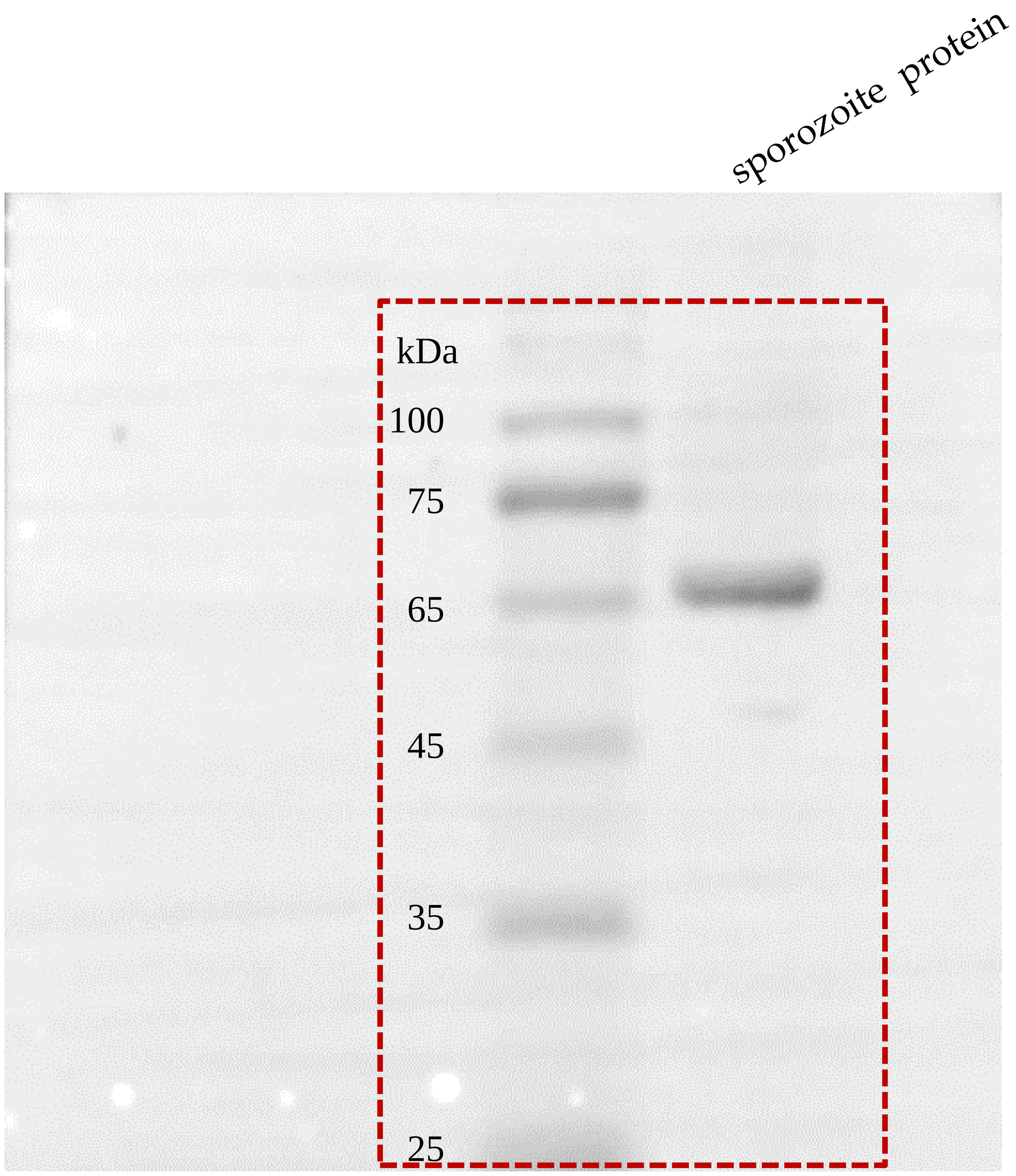

Figure S1B. Investigation of the total sporozoite protein by western blot through the anti-EtROP35 polyclonal antibody. Gel-separated proteins were transferred to polyvinylidene fluoride membranes (0.22 μm pore size; Millipore, Billerica, MA, USA). Western blots were performed using the anti-EtROP35 polyclonal antibody (1:1000). HRP-conjugated anti-mouse IgG (Proteintech Wuhan Sanying, Wuhan, China) served as the secondary antibodies at 1:8000.

Figure S1C

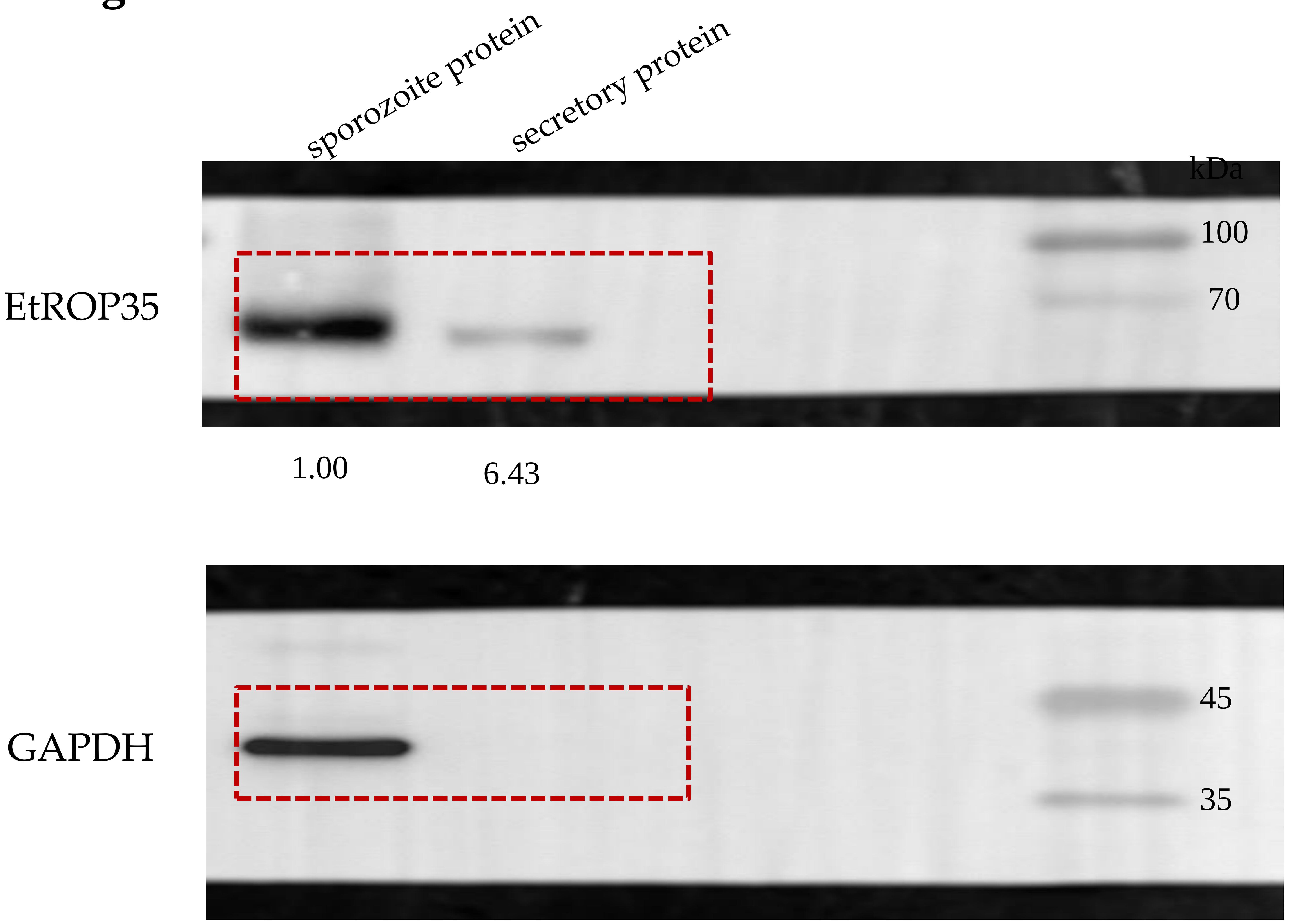

Figure S1C. Western blot analysis of the complete sporozoite protein and the supernatant (secretory protein). Gel-separated proteins were transferred to polyvinylidene fluoride membranes (0.22 μm pore size; Millipore, Billerica, MA, USA). The secretory protein and sporozoite lysate were analyzed using western blot by the anti-EtROP35 polyclonal antibody (1:1000). Anti-GAPDH mouse monoclonal antibody (Solarbio Science & Technology Co., Ltd., Beijing, China) served as the reference control (1:6000), and HRP-conjugated anti-mouse IgG (Proteintech Wuhan Sanying, Wuhan, China) as the secondary antibodies at 1:8000. Densitometry readings/intensity ratio of western blot performed with the Image J 1.8.0 software (National Institutes of Health, Bethesda, MD, USA).

.

Figure S2

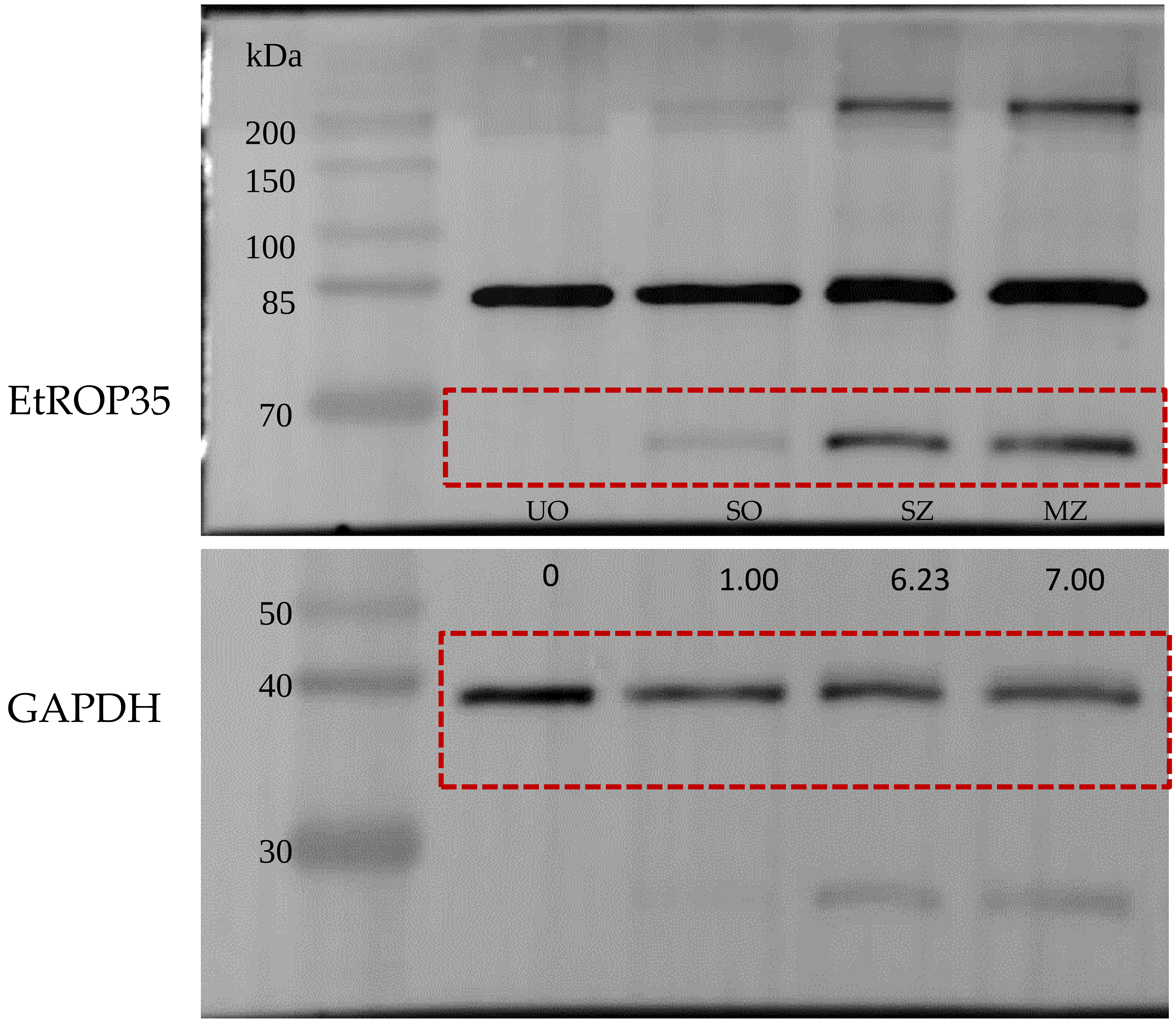

Figure S2. Expression levels of EtROP35 during multiple developmental stages of *E. tenella* analyzed by western blot. Unsporulated oocysts (UO), sporulated oocysts (SO), sporozoites (SZ), merozoites (MZ). Gel-separated proteins were transferred to polyvinylidene fluoride membranes (0.22 μm pore size; Millipore, Billerica, MA, USA). The first antibodies were anti-EtROP35 polyclonal antibody (1:1000) and anti-GAPDH mouse monoclonal antibody (Solarbio Science & Technology Co., Ltd., Beijing, China) at 1:6000. HRP-conjugated anti-mouse IgG (Proteintech Wuhan Sanying, Wuhan, China) served as the secondary antibodies at 1:8000. Densitometry readings/intensity ratio of western blot performed with the Image J 1.8.0 software (National Institutes of Health, Bethesda, MD, USA). GAPDH served as internal reference.

Figure S3

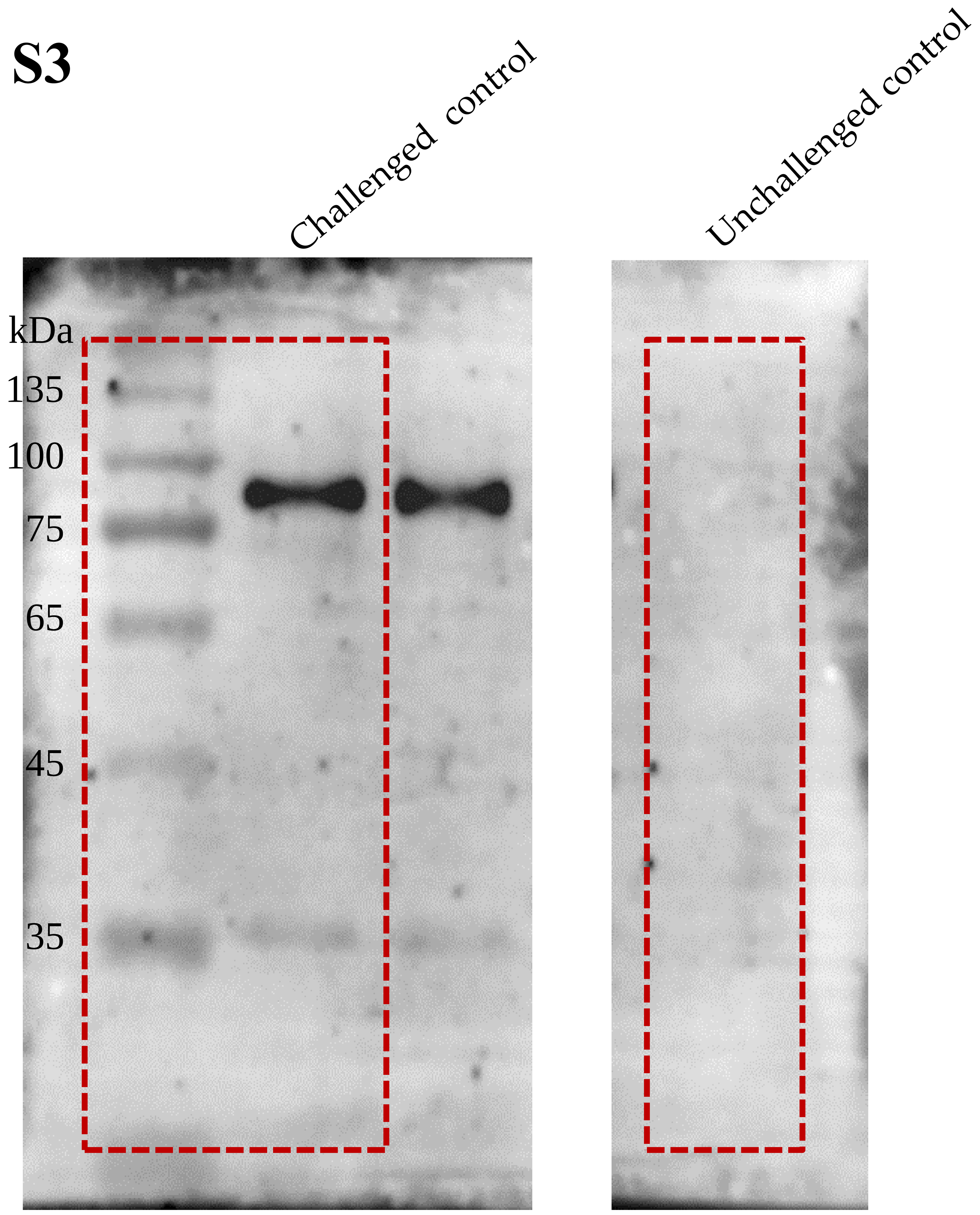

Figure S3. The recombinant EtROP35 recognized by chicken serum from the challenged control group and unchallenged control group. Gel-separated proteins were transferred to polyvinylidene fluoride membranes (0.22 µm pore size; Millipore, Billerica, MA, USA). The first antibodies were chicken serums from the challenged control group (1:1000) and unchallenged control group (1:1000). HRP-conjugated anti-chicken IgG (Solarbio Science & Technology Co., Ltd., Beijing, China) served as the secondary antibodies at 1:1000.
